# Child-friendly family reduces parenting stress in Chinese families: the mediating role of family resilience

**DOI:** 10.3389/fpsyg.2024.1430005

**Published:** 2024-10-21

**Authors:** Amanda Man Ying Chu, Jenny Tsun Yee Tsang, Agnes Tiwari, Helina Yuk, Mike Ka Pui So

**Affiliations:** ^1^Department of Social Sciences and Policy Studies, The Education University of Hong Kong, Tai Po, Hong Kong SAR, China; ^2^School of Nursing, Tung Wah College, Ho Man Tin, Hong Kong SAR, China; ^3^School of Nursing, Hong Kong Sanatorium & Hospital, Happy Valley, Hong Kong SAR, China; ^4^Department of Social Work, The Chinese University of Hong Kong, Shatin, Hong Kong SAR, China; ^5^Department of Information Systems, Business Statistics and Operations Management, The Hong Kong University of Science and Technology, Clear Water Bay, Hong Kong SAR, China

**Keywords:** child-friendly family, parenting stress, family resilience, mediating effect, moderating effect, parent–child relationship, structural equation modeling

## Abstract

The increasing prevalence of parenting stress has significant implications for the psychological well-being of both parents and children. In view of this, our study sought to examine the mediating and moderating role of family resilience in the association between child-friendly family and parenting stress. Our analysis involved a sample of 316 parents who dedicated a minimum of 14 h per week to caring for their children. The parents were invited to complete three validated instruments—the parenting stress index short form (PSI), the family resilience assessment scale (FRAS), and inventory of the child-friendly family (ICF)—to evaluate their level of parenting stress, family resilience, and child-friendly family, respectively. We tested the mediation model by applying structural equation model analysis. It was found that child-friendly family negatively correlated with parenting stress (path coefficient = −0.56, *p* < 0.001). This relationship is mediated by family resilience. That is “child-friendly family” leads to increased “family resilience” (path coefficient = 0.68, *p* < 0.01), which in turn leads to lower “parenting stress” (path coefficient = −0.30, *p* < 0.05). The mediation effect ratio was 26.70%. We used multiple regression analysis to test the moderation model and found that family resilience did not play a moderating role between child-friendly family and parenting stress. This study holds particular significance for two key reasons: Firstly, it elucidates the relationship between child-friendly family, family resilience, and parenting stress, highlighting the potential of creating a child-friendly family to reduce parenting stress through the enhancement of family resilience. Secondly, our findings provide valuable evidence for the development of innovative approaches that effectively and sustainably alleviate parenting stress.

## Introduction

Parenting stress refers to the distress or discomfort that parents experience in fulfilling their parenting responsibilities and roles (Mohammadkhani Orouji, 2021). It is the feeling of being overwhelmed, anxious, or fatigued due to the demands and challenges of raising children (Bornstein, 2002). Recent research indicates that the prevalence of high parenting stress has increased significantly (Aviles et al., 2024). A 2023 survey found that four-in-ten parents (41%) expressed being a parent is tiring and 29% said it is stressful all or most of the time (Minkin and Horowitz, 2023). The rise in parenting stress is possibly due to the increase in single-parent households, children living in poverty, mothers in the workforce, and the living difficulties after the COVID-19 pandemic (Adams et al., 2021).

Parents experiencing high parenting stress have a poor relationship with their children and negatively affect their psychosocial well-being (Robinson and Neece, 2015). Such parents have also been reported to have lower marital quality (Wang et al., 2023). They are also more commonly associated with negative parenting behaviors, such as harsh discipline, hostility, negligence, and child abuse, leading to poorer psychological outcomes for their children and adolescents (Bauch et al., 2022; Schittek et al., 2023). Children raised in high parenting stress families have poorer cognitive skills and learning ability (Zhao et al., 2023). They also demonstrated more social issues, interpersonal difficulties, and internalizing and externalizing problems (Bernier et al., 2022; Shi et al., 2024). Managing parenting stress is therefore important for the well-being of the entire family.

### Bowen family systems theory

The Bowen Family Systems Theory provides a valuable framework for understanding the dynamics between parenting stress, child-friendly family, and the role of family resilience. This theory posits that families operate as emotional units, where individual behaviors and stressors are interconnected (Bowen, 1978). In this context, parenting stress can be seen as a manifestation of relational patterns within the family system (Papero, 1990). A child-friendly family environment, characterized by open communication, emotional support, and healthy boundaries, can help mitigate parenting stress by fostering resilience among family members. Family resilience, as outlined in this theory, serves both as a mediator and moderator, influencing how families respond to stressors. When families possess strong resilience, they are better equipped to manage challenges, thereby reducing the negative impacts of parenting stress on parents (Nichols and Schwartz, 2004). Understanding these relationships is crucial for developing effective interventions that promote healthier family dynamics and enhance overall well-being.

### Child-friendly family and parenting stress

Interventions for reducing parenting stress have been widely investigated. There are interventions with supporting components (e.g., provision of social support to parents by community nurses; van Grieken et al., 2019), interventions with empowerment and skill development components (e.g., mindfulness parenting and life skills training; Burgdorf et al., 2019), and interventions targeting children’s condition (e.g., behavioral intervention for children; Chavez Arana et al., 2020). However, although most of the available interventions may reduce immediate parenting stress, they cannot effectively and sustainably reduce parenting stress (Golfenshtein et al., 2016). Further investigation into new approaches for parenting stress management is necessary. Research findings have indicated that a parenting training program can increase parents’ locus of control, decrease children’s disruptive behaviors, and reduce parenting stress (Moreland et al., 2016). Improving the parent–child relationship through an appropriate method of parenting is a potentially promising approach for managing parenting stress.

The concept of “child-friendly family” has recently gained more attention in research and parenting practice (Chu et al., 2024). A child-friendly family refers to a familial environment characterized by close emotional connections, strong social support, and a focus on children’s overall well-being, allowing them to grow up in a happy, loving, and understanding atmosphere (UNICEF, 2018). Fostering a child-friendly family environment promotes a trusting parent–child relationship. In this approach, parents establish reasonable boundaries and consequences, encourage open communication and provide their children with understanding and acceptance. By adopting child-friendly parenting, parents can also develop positive character traits in themselves, such as patience, kindness, empathy, joy, and gentleness. They learn to handle meltdowns with composure, reject spanking as an effective form of discipline, resist victimhood in parenthood, and maintain safe and respectful family relationships. It has been reported that child-friendly parenting can inhibit problematic behavior in children (Pinquart, 2017) and that better child behavior is associated with lower parenting stress (Davis and Neece, 2017).

### Family resilience as a mediator

Family resilience is a social protective factor that gives entire families and individual family members the ability to recover from adversity stronger and more resourceful (Sixbey, 2005). It contains family relationship-enhancing components, including effective communication, problem-solving strategies, and access to social and economic resources (Walsh, 2002). High resilience families exhibit better parent–child relationships, less vulnerability to challenges, and a greater ability to provide a secure environment to all family members (Herbell et al., 2020). Parents with higher family resilience are more likely to have a better psychological status (Aivalioti and Pezirkianidis, 2020) whereas parenting stress has been found to be negatively correlated with family resilience (Silva et al., 2021).

**Figure 1:**
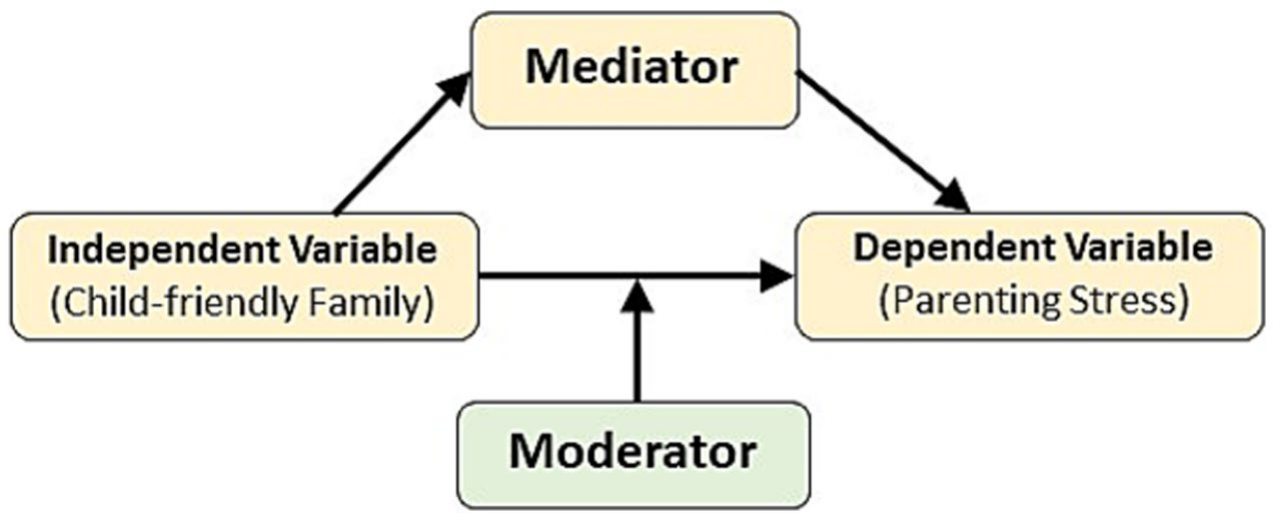
Relationship between mediator, moderator, independent variable (child-friendly family), and dependent variable (parenting stress).

In this context, family resilience appears to act as a mediator. As shown in Figure 1, a mediator is a variable that lies on the causal pathway between an independent variable (child-friendly family) and a dependent variable (parenting stress) (Morrow et al., 2022). That is if “family resilience” is a mediator, it will correlate with both “child-friendly family” and “parenting stress,” as well as mediating the effect of “child-friendly family” on “parenting stress.” Thus, the hypothesis (H1) is that family resilience mediates the causal relationship between child-friendly family and parenting stress.

*H1: Family resilience is a mediator of the effect of child-friendly family on reducing parenting stress.*

### Family resilience as a moderator

Instead of being a mediator, family resilience could be a moderator of the relationship between child-friendly family and parenting stress. A moderator can modify the relationship between the independent variable (child-friendly family) and the dependent variable (parenting stress) (Figure 1) (Morrow et al., 2022). It does not lie on the causal pathway but interacts with the independent variable in a way that influences the outcome (Baron and Kenny, 1986). That is if “family resilience” is a moderator, it can affect the strength or direction of the effect of “child-friendly family” on “parenting stress.”

Previous studies have investigated the moderating effect of family resilience on children’s behavior. The findings indicate that family resilience has a negative moderating effect on negative parenting and adolescents’ problematic behaviors (Zhuo et al., 2022). Because family resilience has been reported to be a moderator of the relationship between negative parenting and adolescents’ problematic behaviors, and the problematic behaviors of children have been found to correlated with parenting stress, we posit the hypothesis:

*H2: Family resilience is a moderator of the effect of child-friendly family on reducing parenting stress.*

## Materials and methods

### Participants

This study was conducted with 316 parents. Inclusion criteria for this study encompassed parents who provided care for their children for a minimum of 14 h per week. Participants were required to be biological parents of children aged 0 to 18 years. Exclusion criteria included parents who were not the primary caregivers or who were unable to communicate effectively in the survey language, as well as those who had a significant mental health diagnosis that could impede their ability to provide accurate responses regarding their parenting experiences.

The demographic characteristics of the participants are shown in Table 1. The majority of the participants were female (97.2%). Nearly three quarters of the participants were in either the 31–40 (44.0%) or 41–50 (30.7%) age group. Regarding educational level, most of the participants had received high school or tertiary education (89.9%). Most of the participants had no salaried job (79.4%). Slightly more than three quarters of the participants were married or cohabitating (77.5%). The highly diversified backgrounds of the participants provided a representative sample for this study and reduced potential biases due to the influence of socioeconomic background.

**Table 1:** Demographic characteristics of participants.

	*n*	%
Gender		
Male	9	2.8
Female	307	97.2
Age		
>20 – <=30	31	9.8
31–40	139	44.0
41–50	97	30.7
> = 51	49	15.5
Educational level		
Primary school	32	10.1
Junior high school	99	31.3
Senior high school	139	44.0
Tertiary or above	46	14.6
Employment status		
At work	65	20.6
Not at work	251	79.4
Place of birth		
In Hong Kong	113	35.8
Outside Hong Kong	203	64.2
Marital status		
Single	12	3.8
Married/Cohabiting	245	77.5
Divorced/Separated/Spouse passed away	59	18.7

### Procedures

The data were collected in 2019 from a nonprofit organization that has provided integrated family and community services in Hong Kong for more than 30 years. The registered social workers of the organization recruited parents who provided care to their children for at least 14 h per week to participate in the study. Before interviewing the potential participants, the social workers explained the study to them and obtained their informed consent. All the participants took part on a voluntary basis. The participants were asked to provide their demographic characteristics and complete three paper-and-pencil questionnaires: the Parenting Stress Index–Short Form (PSI; Rivas et al., 2021), the Family Resilience Assessment scale (FRAS; Chu et al., 2022), and the Inventory of the Child-Friendly Family (ICF; Chan and Chen, 2019).

### Measures

#### Parenting stress index–short form

The PSI is a commonly used measure of parenting stress in clinical and research contexts (Rivas et al., 2021). It contains 36 items with three subscales—parental distress, parent–child dysfunctional interaction, and difficult child. Each of the 36 items in the PSI is measured on a five-point Likert scale ranging from 1 (strongly agree) to 5 (strongly disagree), with a total score ranging from 36 to 180. A higher total PSI score represents a lower level of parenting stress. Abidin (1995) reported Cronbach’s alpha coefficients of 0.91 for PSI total score.

#### Family resilience assessment scale

The FRAS has been widely used to determine family resilience in various contexts (Gardiner et al., 2019). A validated Chinese version of the FRAS (Chu et al., 2022) was adopted in this study. The Chinese FRAS contains 42 items with five subscales: ability to make meaning of adversity, family communication and problem-solving, maintaining a positive outlook, family spirituality, and utilizing social and economic resources. Each item is measured on a four-point Likert scale ranging from 1 (strongly disagree) to 4 (strongly agree). A higher total FRAS score represents a higher level of family resilience. The Chinese FRAS demonstrated adequate concurrent validity and internal consistency (*α* = 0.724–0.963).

#### Inventory of the child-friendly family

The ICF was developed to guide parents and other stakeholders to build a child-friendly family (Chan, 2008). It was validated in a Chinese student population (Chan and Chen, 2019) and contains 18 items with two subscales: psychological support and positive discipline strategies, and care and protection. The ICF was originally designed to gain responses from children or adolescents. In the current study, we measured the child friendliness level of a family on the basis of the parents’ perspectives. Therefore, we modified the measurement items in the ICF by swapping the subject and object in the items. For example, the item “My parents satisfy my basic needs” was changed to “I satisfy my child’s basic needs.” In the ICF, each item is measured on a four-point Likert scale ranging from 1 (strongly disagree) to 4 (strongly agree). A higher total score of the ICF indicates that the family is more child friendly. The Cronbach’s alphas values of the two subscales of “psychological support and positive discipline strategies” and “care and protection” in the currency study were 0.766 and 0.824, respectively. The internal consistency level is acceptable because the values of Cronbach’s alphas are equal to or greater than 0.7 (Tavakol and Dennick, 2011).

#### Data analysis

Structural equation model (SEM) analysis was applied to investigate the relationship between child-friendly family, parenting stress, and family resilience, as well as the mediating role of family resilience between child-friendly family and parenting stress. The SEM analysis was conducted using the AMOS 26 software, along with fit indices calculation. The model fit was determined by various fit indices, including goodness-of-fit statistic (GFI), normed-fit index (NFI), root mean square residual (RMR), and root mean square error of approximation (RMSEA). The recommended cut-off for a good model fit is GFI > = 0.90, NFI > 0.90, RMR < 0.08, and RMSEA <0.08 (Hu and Bentler, 1999).

Multiple regression analysis was applied to examine the moderation effect of family resilience on parenting stress regulation by child-friendly family. The analysis was conducted using the SPSS 26 software.

## Results

### Descriptive statistics and correlations

Table 2 presents the means, standard deviations, and Pearson correlation coefficients for PSI, FRAS, and ICF. PSI was positively correlated with FRAS (0.565, *p* < 0.01) and ICF (0.599, *p* < 0.01), suggesting that higher levels of parenting stress were associated with lower levels of family resilience and lower levels of child friendliness in a family. FRAS was positively correlated with ICF (0.537, *p* < 0.01), indicating that family resilience was positively associated with child-friendly family.

**Table 2:** Means, standard deviations, and bivariate correlations between the results of PSI, FRAS, and ICF.

			Correlations		
	Mean	SD	1	2	3
PSI	112.85	22.58	1		
FRAS	115.82	24.84	0.565**	1	
ICF	52.41	5.642	0.599**	0.537**	1

PSI, parenting stress index; FRAS, family resilience assessment scale; ICF, inventory of the child-friendly family. **p < 0.01.

### Family resilience as a mediator between child-friendly family and parenting stress

**Figure 2:**
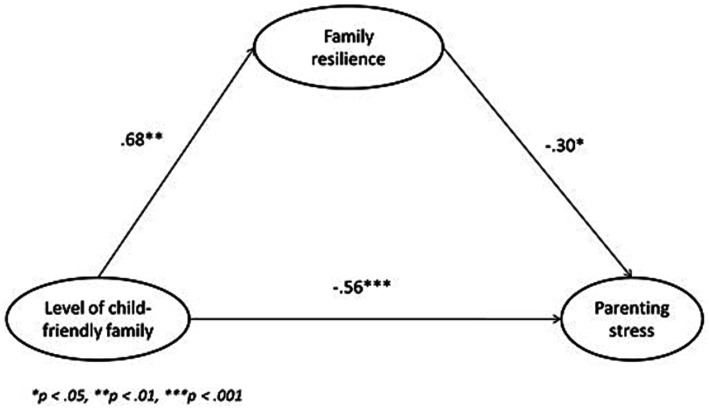
The mediation model of the effect of family resilience on child-friendly family and parenting stress.

The structural equation model of the three variables—child-friendly family, family resilience, and parenting stress—is presented in Figure 2. In this model, all the path coefficients were significant, χ^2^ = 98.157, *df* = 41, and the fit indices were satisfactory (GFI = 0.949, NFI = 0.925, RMR = 0.014 and RMSEA = 0.053).

Child-friendly family demonstrated a negative correlation with parenting stress (path coefficient = −0.56, *p* < 0.001). The negative path coefficient indicated that a higher level of child friendliness in a family leads to a lower level of parenting stress. Moreover, child-friendly family demonstrated a positive correlation with family resilience (path coefficient = 0.68, *p* < 0.01), while family resilience demonstrated a negative correlation with parenting stress (path coefficient = −0.30, *p* < 0.05). The results suggested that a higher level of child friendliness in a family brings about a higher level of family resilience, which in turn leads to a lower level of parenting stress.

The SEM model supported the existence of direct relationships between child-friendly family, family resilience, and parenting stress. It also indicated that family resilience was a mediator between child-friendly family and parenting stress, supporting hypothesis H1. The mediation effect ratio was calculated as follows:


$$\mathrm{Mediation}\;\mathrm{effect}\;\mathrm{ratio}=\frac{\mathrm{Standardized}\;\mathrm{indirect}\;\mathrm{effect}\;\mathrm{value}\;}{\mathrm{standardized}\;\mathrm{total}\;\mathrm{effect}\;\mathrm{value}}$$

The standardized indirect effect value of child-friendly family→family resilience→parenting stress was 0.68 x − 0.30 = −0.204. The standardized total effect value of child-friendly family on parenting stress was (−0.56) + (−0.204) = −0.764. On the basis of the two values, the mediation effect ratio was calculated as 0.204/0.764*100% = 26.70%. The mediation effect ratio showed that the mediation effect from family resilience contributed to over one quarter of the total effect of child-friendly family on parenting stress.

### Moderation effect of family resilience on child-friendly family and parenting stress

Multiple regression analysis was applied to examine the moderation effect of family resilience on child-friendly family and parenting stress. There were two models in this analysis, which are presented in Table 3. In model 1, family resilience and child-friendly family were significant predictors of parenting stress (*p* < 0.001), which was consistent with the findings of the SEM model. In model 2, an interaction term of child-friendly family and family resilience (ICF × FRAS) was added to the list of independent variables. After adding the interaction term (ICF × FRAS), family resilience and child-friendly family remained significant predictors of parenting stress (*p* < 0.001). However, the interaction term was not significant in the model. The F-change in model 2 was 0.50 (*p* = 0.824), which implied that the change in R-square was not significant. The results showed that there was no moderation effect of family resilience on the relationship between child-friendly family and parenting stress and thus hypothesis H2 was rejected.

**Table 3:** Multiple regression analysis for the effect of family resilience on the relationship between child-friendly family and parenting stress.

	Predictor	*B*	Sig. of predictor	*F* change	Sig. of *F* change
Model 1				124.095	0.000
	Family resilience	0.519	0.000		
	Level of child friendliness	1.667	0.000		
	Constant	−34.640	0.000		
Model 2				0.050	0.824
	Family resilience	0.518	0.000		
	Level of child friendliness	1.667	0.000		
	ICF × FRAS	0.002	0.824		
	Constant	−34.593	0.000		

## Discussion

In this study, we found out that child-friendly family is negatively correlated with parenting stress. It is also found that family resilience acts as a mediator, but not a moderator, among the relationship between child-friendly family and parenting stress.

### Relationship between child-friendly family and parenting stress

Our findings indicated that child-friendly family demonstrated a negative correlation with parenting stress as shown in Figure 2 (path coefficient = −0.56, *p* < 0.001), suggesting a lower level of child friendliness in a family associates with a higher level of parenting stress. It is consistent with the other findings that indicates a poor parent–child relationship is commonly associated with negative parenting, such as the use of physical coercion, verbal hostility, and punitive action. Children from families with negative parenting are more frequently associated with problematic behaviors, such as agitation. In the face of problematic behaviors, stressful parents tend to increase their use of negative parenting. However, rather than improving children’s behavior, the entire family get trapped in a vicious circle of deteriorating parent–child relationships and intensifying parenting stress (Bauch et al., 2022; Schittek et al., 2023). The results of this study indicate the importance of promoting child-friendliness in the family to reduce maternal parenting stress.

### Family resilience acts as a mediator

This study identified family resilience as a mediator between child-friendly family and parenting stress. A higher level of child friendliness in a family increases family resilience, which in turn reduces parenting stress. The mediation effect ratio was 26.70%, indicating that the mediation effect from family resilience contributed to over one quarter of the total effect of child-friendly family on parenting stress. This is consistent with Walsh’s family resilience theory (Walsh, 2002) that indicates the correlation between family resilience and parenting stress. The research findings of the current study have also confirmed the findings of the previous studies on the relationship between family resilience and parenting stress. According to the study of Kim et al., 2020, family resilience is negatively associated with maternal parenting stress that parents with high levels of family resilience had 15–18% lower probability for parenting stress. Studies on military parents revealed that improvement in key family resilience processes is predictive of an improvement in parents’ mental health, including a reduction in the distress and stress of parenting (Saltzman et al., 2016). Moreover, family resilience stabilizes the emotions of parents. Previous studies conducted among 437 parents provided evidence that emotionally distressed parents have a more negative perception of their children (Laufer and Bitton, 2023). On the contrary, parents who are free from emotional distress may have a better perception of their children and evaluate their behavior in a more positive way.

### Family resilience is not a moderator

According to our results, family resilience is not a moderator in the relationship between child-friendly family and parenting stress. This implies that the direct impact of child-friendly family on parenting stress is stable, and the strength and direction of the relationship would not be affected by family resilience. However, previous studies which collected responses from teenagers found that family resilience has a negative moderating effect on the relationship between negative parenting and problematic behaviors (Sun et al., 2015). The difference in results may suggest that children and parents have different views on the same family issue.

### Implications

Recognizing the role of the child-friendly family in enhancing family resilience and reducing parenting stress in the Chinese community is a particularly important finding. In traditional Chinese culture, filial piety is a virtue. Absolute obedience to and respect for parents is perceived to be mandatory. Conventionally, Chinese parents think they have “ownership” and “control” of their children (Cao and Wang, 2020). Studies have revealed that many Chinese parents do not regard using threatening or humiliating methods to control children as inappropriate and do not understand that such methods are harmful to the parent–child relationship (Li, 2019; Smith, 2016). Our findings, however, revealed that promoting the child-friendly family is beneficial to Chinese families. The adoption of child-friendly parenting among Chinese family not only does no harm to the filial piety-oriented tradition but also effectively enhances family resilience and reduces parenting stress.

Further investigation of other potential mediating and moderating factors between child-friendly family and parenting stress will provide a better understanding of how the child-friendly family enhances family resilience and reduces parenting stress. In the future, society will continue to develop at an unprecedented pace. People will need to adapt to new and unexpected changes frequently, thus increasing parenting stress. Promoting the child-friendly family is especially important in empowering individuals, as well as their families, to become more adaptable to changes through higher family resilience and lower parenting stress.

## Limitations and future research

The results in the current study were based on a survey conducted in Hong Kong. To generalize the results more broadly, further research is needed. In addition, the significance of the research model in the current study depends on the reliability of self-reports. To minimize response bias, all participants had a clear understanding of the nature of the study from experienced social workers, and we used diverse participants. A pretest was also conducted before the actual survey to ensure the quality of the study. While the current study included both mothers and fathers, the sample was predominantly consisting of mothers. It is a common phenomenon of having more mothers than fathers participate in parenting studies (Davison et al., 2016). However, this unequal representation may limit the ability to make strong claims about the broader population of parents. Similarly, uneven employment status distribution among participate may also limit the generalization of the results. Further study should aim to obtain a more balanced sample with comparable proportion of mothers and fathers, and different employment status. In addition, the absence of confounding variables in our research model may limit the generalizability of our findings. Further research could benefit from incorporating relevant confounding variables to provide a more comprehensive understanding of the interactions. In the current study, we used parents as our target respondents. However, some previous studies have used children as target respondents to learn about parenting or family issues. Further research is recommended to investigate why and how parents and children perceive the same parenting or family issue differently.

## Data Availability

The raw data supporting the conclusions of this article will be made available by the authors, without undue reservation.
